# Ibuprofen and Palmitic Acid–Based Solvent Exchange–Induced In Situ Forming Matrices With Gentian Violet for Oropharyngeal Candidiasis and Periodontitis Treatment

**DOI:** 10.1155/adpp/1580663

**Published:** 2026-07-15

**Authors:** Thawatchai Phaechamud, Panadda Phattanawasin, Setthapong Senarat, Utsana Puapermpoonsiri, Pimjai Pimbaotham, Siriporn Jungsuttiwong, Sai Myo Thu Rein, Kritamorn Jitrangsri

**Affiliations:** ^1^ Division of Industrial Pharmacy, Faculty of Pharmacy, Silpakorn University, Nakhon Pathom, 73000, Thailand, su.ac.th; ^2^ Division of Pharmaceutical Chemistry and Technology, Faculty of Pharmaceutical Sciences, Ubon Ratchathani University, Ubon Ratchathani, 34190, Thailand, ubu.ac.th; ^3^ Department of Chemistry and Center of Excellence for Innovation in Chemistry, Ubon Ratchathani University, Ubon Ratchathani, 34190, Thailand, ubu.ac.th; ^4^ Department of Pharmacognosy, University of Pharmacy, Mandalay, Myanmar; ^5^ Department of Industrial Pharmacy, School of Pharmacy, Walailak University, Nakhon Srithammarat, 80160, Thailand, wu.ac.th; ^6^ Biomass and Oil Palm Center of Excellence, Walailak University, Tha Sala, Nakhon Si Thammarat, 80160, Thailand, wu.ac.th

**Keywords:** antimicrobial activity, density functional theory, gentian violet, ibuprofen, *in situ* forming matrix, palmitic acid

## Abstract

**Background:**

Gentian violet (GV) is a broad‐spectrum antimicrobial agent with documented efficacy against oropharyngeal candidiasis and periodontal pathogens, but its clinical use is limited by poor local retention. In situ forming matrices (ISMs) offer a promising strategy for sustained, localized drug delivery.

**Objectives:**

This study aimed to develop and evaluate GV‐loaded ISMs using ibuprofen (IBU) and palmitic acid (PA) as dual‐function matrix‐forming agents in DMSO and NMP solvents for the localized treatment of oropharyngeal candidiasis and periodontitis.

**Methods:**

ISM formulations were prepared by simple mixing and characterized for viscosity, rheological behavior, injectability, mechanical properties, and in situ matrix formation. In vitro drug release of GV and IBU was quantified by a validated simultaneous HPLC method using an ACE C18 column with UV detection at 590 and 222 nm, respectively. Drug‐release kinetics were modeled using zero‐order, first‐order, Higuchi, Korsmeyer–Peppas, and Peppas–Sahlin models. Antimicrobial activity against *Staphylococcus aureus*, *Candida albicans*, *C. tropicalis*, and *Porphyromonas gingivalis* was assessed by agar diffusion over 15 days. Molecular interactions were investigated using density functional theory (DFT) calculations at the B3LYP‐D3BJ/6‐31G(d,p) level.

**Results:**

All formulations showed Newtonian‐flow behavior and acceptable injectability (0.78–1.50 N). GV and IBU release followed the Peppas–Sahlin model, with NMP‐based systems releasing GV and IBU faster due to more porous matrix architecture, while DMSO‐based systems formed denser matrices with slower release. All GV‐containing formulations maintained antimicrobial inhibition zones for up to 15 days. DFT calculations revealed strong GV–IBU (−1.63 eV) and GV–PA (−1.53 eV) binding energies, supporting sustained drug entrapment.

**Conclusions:**

GV‐loaded IBU/PA‐based ISMs demonstrate sustained antimicrobial efficacy for up to 15 days with solvent‐dependent release behavior. These systems show potential as localized, single‐injection therapies for oral infections. Stability evaluation and in vivo biocompatibility studies are identified as necessary future steps.

## 1. Introduction

**FIGURE 1:**
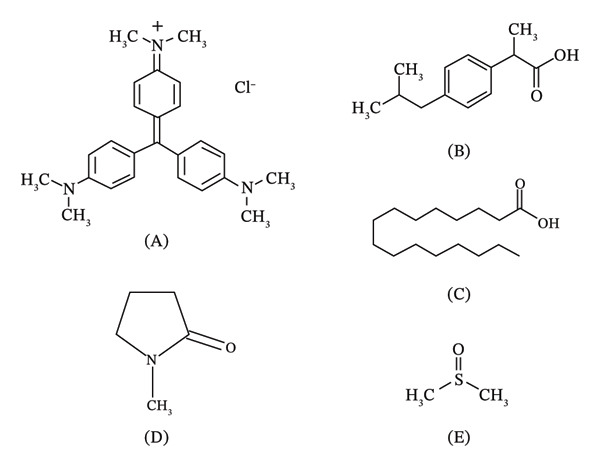
Chemical structures of gentian violet (A), ibuprofen (B), palmitic acid (C), and *N*‐methyl‐2‐pyrrolidone (D), and dimethyl sulfoxide (E).

Gentian violet (GV) (Figure 1A), also known as methylrosaniline chloride, is a triphenylmethane dye recognized for its vivid purple color and widely used in staining applications [1]. With a molecular formula of C_25_H_30_ClN_3_ and a molar mass of 407.98 g/mol, GV is water‐ and alcohol‐soluble [2], facilitating its incorporation into various formulations for topical and therapeutic use. Its cationic nature enhances its binding affinity to negatively charged surfaces, such as microbial cell walls [3]. GV has broad‐spectrum antimicrobial activity, demonstrating efficacy against multidrug‐resistant bacteria, including *Staphylococcus aureus* and *Pseudomonas aeruginosa* [4], as well as antifungal effects against *Candida albicans* by inhibiting germ tube formation and adherence [5]. Clinical studies have shown that GV is safe and effective in treating oropharyngeal candidiasis (OPC) in HIV‐1–infected adults, with comparable efficacy to nystatin and no significant mucosal staining at therapeutic concentrations [6, 7]. Additionally, GV exhibits low toxicity in local applications, with an LD_50_ ranging between 2.5 and 5.0 g/kg in animal studies [8]. Unlike conventional antifungals such as fluconazole and nystatin, or periodontal antiseptics such as chlorhexidine, GV exerts broad‐spectrum antimicrobial activity through multiple nonspecific mechanisms including the disruption of fungal cell wall integrity and inhibition of germ tube formation, rendering resistance development unlikely [9]. Its cationic and aromatic nature further confers strong molecular affinity toward the hydrophobic IBU/PA matrix components, making GV particularly well‐suited for sustained, localized delivery within a solvent exchange–induced ISM platform.

OPC is a common fungal infection caused primarily by *C. albicans*, affecting the oral mucosa, throat, and tongue. It frequently occurs in immunocompromised individuals, such as those with HIV/AIDS, diabetes, or undergoing chemotherapy, as well as denture wearers and prolonged antibiotic or corticosteroid users [10]. Symptoms include white plaques, erythema, pain, and dysphagia, significantly impacting patients′ quality of life [11]. Conventional treatments, such as azole antifungals (e.g., fluconazole) and polyenes (e.g., nystatin), face challenges such as drug resistance, poor mucosal adhesion, and frequent dosing requirements, necessitating the development of more effective and sustained‐release formulations [12, 13].

Periodontitis is a chronic inflammatory disease characterized by the destruction of periodontal tissues, including the gingiva, periodontal ligament, and alveolar bone, primarily due to dysbiotic bacterial biofilms, particularly, *Porphyromonas gingivalis* and *Aggregatibacter actinomycetemcomitans* [14, 15]. It can lead to tooth loss and systemic complications with improper treatment. Current treatments involve mechanical debridement, antibiotics (e.g., metronidazole and doxycycline), and antiseptics (e.g., chlorhexidine) [16], but these often fail to fully eradicate biofilms or prevent recurrence, highlighting the necessity for improved therapeutic strategies. Local drug delivery systems have emerged as promising alternatives, allowing for high concentrations of antimicrobials to be delivered directly to periodontal pockets, enhancing tissue penetration and sustaining drug release over longer periods [17, 18]. The development of novel drug delivery systems that enhance antimicrobial penetration and sustain drug release could significantly improve therapeutic outcomes for OPC and periodontitis.

In situ forming matrices (ISMs) have emerged as a promising approach for site‐specific drug delivery due to their ability to form a solid‐like matrix upon injection into the body, enhancing localized therapeutic efficacy while minimizing systemic exposure. The primary mechanism of ISMs involves phase inversion, wherein a drug‐loaded polymeric solution transforms into a gel or solid form through solvent exchange once it encounters bodily fluids. This mechanism allows for sustained drug‐release profiles that can be tailored based on the polymer characteristics and formulation specifics. For instance, systems utilizing poly(lactide‐co‐glycolide) (PLGA) have highlighted their biodegradability and ability to control drug release through polymer precipitation after immersion in aqueous environments [19, 20]. Another interesting variation in ISMs involves beta‐cyclodextrin, which improves aqueous solubility and enhances drug‐release profiles by encapsulating drugs in a controlled manner using the phase inversion mechanism [21]. ISMs present a versatile platform for applications ranging from depot delivery systems to antimicrobial treatment for periodontitis, employing various solvents and carriers that conform to the specific requirements of the therapeutic agents used [22–24].

Ibuprofen (IBU) (Figure 1B), a well‐established nonsteroidal anti‐inflammatory drug (NSAID), has been widely used for managing pain and inflammation in periodontal and oral mucosal diseases [25]. In addition to its anti‐inflammatory and analgesic effects, IBU demonstrates weak antibacterial and biofilm‐disrupting activities, particularly against oral pathogens such as *P. gingivalis* and *S. mutans* [26]. Its incorporation into ISM formulations not only supports structural stability of the hydrophobic matrix but also provides synergistic therapeutic benefits by alleviating inflammation associated with OPC and periodontitis.

Palmitic acid (PA) (Figure 1C), a saturated fatty acid widely present in biological membranes and dietary sources, has been increasingly explored in pharmaceutical formulations due to its excellent biocompatibility, biodegradability, and safety profile [27]. In ISMs, PA serves as a hydrophobic matrix‐forming agent that undergoes solvent exchange upon contact with aqueous environments, leading to phase inversion and depot formation. Its lipophilic nature restricts water penetration and drug diffusion, thereby sustaining release kinetics of incorporated therapeutics [28]. The use of PA as a natural excipient also reduces the risk of adverse reactions and aligns with the growing demand for lipid‐based, patient‐friendly drug delivery platforms.

*N*‐methyl‐2‐pyrrolidone (NMP) (Figure 1D), a commonly used solvent in ISMs, is a very strong solubilizing agent, capable of dissolving a wide range of substances, including pharmaceuticals and polymers [29]. NMP forms strong hydrogen bonds with water and alcohol, allowing it to be miscible in the aqueous solvent [30]. Dimethyl sulfoxide (DMSO) (Figure 1E), a clear odorless liquid with molecular formula of (CH_3_)_2_SO [31], is an aprotic, polar solvent that does not donate hydrogen atoms but stabilizes positive charges through its oxygen atom, allowing its miscibility with both water and organic solvents [32, 33]. It has low toxicity and good safety profile with the only solvent FDA‐approved for the treatment of interstitial cystitis and for use as a preservative for organ transplant [34]. They have been utilized as the vehicles of solvent exchange–induced ISMs [22–24].

The combination of GV, PA, and IBU in a solvent exchange–induced ISM presents a multifunctional strategy such as GV offers potent antimicrobial effects, PA ensures controlled release through matrix formation, and IBU addresses inflammation and pain, collectively enhancing treatment efficacy and patient compliance. Therefore, the present study focuses on the development and evaluation of GV‐loaded PA and IBU‐based IFMs using NMP or DMSO as solvents for the localized treatment of OPC and periodontitis.

## 2. Materials and Methods

### 2.1. Materials

GV (Batch No. J‐193779, DC Finechem, Cornella de Llobregat, Spain, was purchased and used as received. IBU (Lot No. 4000/1101/18/A‐0150B) was purchased from P.C. Drug Center Co., Ltd., Bangkok, Thailand. PA (Batch No. 668F180902D) was obtained from Pacific Oleochemicals, Pasir Gudang, Malaysia. DMSO (≥ 99.9%) and *N*‐methyl‐2‐pyrrolidone (NMP, ≥ 99%) were obtained from QReC (New Zealand). Agarose powder was obtained from Vivantis (Malaysia). All chemicals were of analytical grade and used without further purification. Microbial strains *S. aureus* ATCC 6538, *C. albicans* ATCC 10231 and *C. tropicalis* TISTR 5306 were purchased from the Department of Medical Sciences, Ministry of Public Health, Nonthaburi, Thailand. *P. gingivalis* ATCC 33277 (MicroBiologics Inc., St. Cloud, MN, USA) was purchased from Thai Can Biotech Co., Ltd., Bangkok, Thailand. Tryptic soy agar (TSA) and tryptic soy broth (TSB) (Difco, Detroit, MI, USA), along with sheep blood agar (Department of Medical Science, Ministry of Public Health, Mueang District, Nonthaburi, Thailand), were utilized as culture media for antibacterial evaluation. Sabouraud dextrose agar (SDA) and Sabouraud dextrose broth (SDB) (Difco, Detroit, MI, USA) were used for assessing antifungal activity.

### 2.2. Preparation of GV‐Loaded ISM Formulations

GV‐loaded ISMs were formulated using a simple mixing technique, in which the components were continuously stirred with a magnetic stirrer (Benchmark H3770‐HS‐E Digital Hotplate Stirrer, Benchmark Scientific Inc., Sayreville, NJ, USA) at room temperature. Firstly, PA and IBU were first dissolved in either DMSO or NMP under magnetic stirring at 500 rpm until a clear solution was obtained at various ratios according to Table 1. GV was subsequently incorporated into the mixtures at predetermined concentrations with continuous stirring until homogeneous formulations were achieved. The prepared formulations were stored in tightly closed glass vials at room temperature until further use. Control formulations without GV were prepared under identical conditions for comparison.

**TABLE 1:** Compositions of GV‐loaded ISM formulations in DMSO and NMP.

Formulation	Concentration (%w/w)				
	Gentian violet	Ibuprofen	Palmitic acid	DMSO	NMP
GD	1	—	—	99	—
GI40D	1	40	—	59	—
GI50D	1	50	—	49	—
GP40D	1	—	40	59	—
GP50D	1	—	50	49	—
GI20P20D	1	20	20	59	—
GI25P25D	1	25	25	49	—
I50D	—	50	—	50	—
P50D	—	—	50	50	—
DMSO	—	—	—	100	—

GN	1	—	—	—	99
GI40N	1	40	—	—	59
GI50N	1	50	—	—	49
GP40N	1	—	40	—	59
GP50N	1	—	50	—	49
GI20P20N	1	20	20	—	59
GI25P25N	1	25	25	—	49
I50N	—	50	—	—	50
P50N	—	—	50	—	50
NMP	—	—	—	—	100

### 2.3. Physicochemical Characterization

#### 2.3.1. Viscosity and Rheological Behavior

Viscosity was determined using a Brookfield DV3T rheometer (Brookfield Engineering, Middleboro, MA, USA) at 25°C. The rheological properties of ISM formulations were determined using a controlled‐stress rheometer (RM 100 CP2000 plus, Lamy Rheology Instruments Co., Champagne‐au‐Mont‐d’Or, France) equipped with a cone–plate geometry (diameter 50 mm, cone angle 1°, gap 0.05 mm). Measurements were conducted at 25 ± 0.5°C and 37 ± 0.5°C. Shear stress (0.1–100 s^−1^) was applied, and viscosity values were recorded at 15‐s interval. Flow curves were obtained by plotting shear stress against the shear rate.

#### 2.3.2. Injectability and Mechanical Properties

Injectability was assessed using a texture analyzer (TA.XTplus, Stable Micro Systems, Surrey, UK) fitted with a 5‐kg load cell. Each formulation (1 mL) was loaded into a 1‐mL disposable syringe fitted with an 18‐gauge needle. The probe was programmed to depress the plunger at a speed of 1 mm/s, and the maximum expelling force was recorded (*n* = 3). Mechanical properties (hardness, adhesiveness, cohesiveness) were measured using the compression mode of the texture analyzer with a cylindrical probe (diameter 10 mm) at a compression speed of 1 mm/s.

### 2.4. Matrix Formation Behavior

#### 2.4.1. In PBS Solution

Matrix formation in an aqueous environment was evaluated by injecting 1 mL of each ISM formulation into 5 mL of PBS (pH 6.8) maintained at 37°C in glass vials. The transformation from liquid to the solid/gel matrix by solvent exchange–induced phase inversion was visually monitored at time intervals (1, 5, 10, and 20 min).

#### 2.4.2. In Agarose Wells

Agarose gels (0.6% w/v in PBS) were cast into 90‐mm petri dishes. Wells of 7 mm diameter were created using a plastic straw. Aliquots of 150 μL ISM formulations were introduced into wells, and phase inversion was observed under a stereomicroscope (SMZ745T, Nikon, Tokyo, Japan) at × 20 magnification at 1, 5, 10, 15, 20 and 30 min).

### 2.5. *In Vitro* Drug Release

The release of GV and IBU was investigated using a modified cup diffusion method. Cylindrical glass cups (internal diameter 10 mm, height 10 mm) were filled with 0.4 g of ISM formulation and placed in 50 mL of PBS (pH 6.8) at 37 ± 0.5°C under constant agitation (100 rpm) in a shaking water bath (Julabo SW23, Seelbach, Germany). At predetermined time intervals (0.5–120 h), 1‐mL aliquots were withdrawn and replaced with fresh PBS. GV and IBU was quantified by a validated high‐performance liquid chromatography (HPLC) method. Chromatographic separation was carried out at room temperature on an ACE C18 column (4.6 × 250 mm, 5 μm). The mobile phase comprised 0.2% (v/v) sulfuric acid and acetonitrile (40:60, v/v) and was delivered isocratically at a flow rate of 1.0 mL min^−1^. The injection volume was 5 μL. A single HPLC method was used for simultaneous quantification of both GV (590 nm) and IBU (222 nm) from the same sample, enabling concurrent estimation of dual‐drug release without separate analytical runs. The cumulative % IBU and GV release amounts from ISMs were calculated (*n* = 3). To elucidate the drug‐release mechanism, the dissolution data were analyzed using various kinetic models, including the zero‐order, first‐order, Higuchi’s, Korsmeyer–Peppas, and Peppas–Sahlin models. The optimal model was identified based on the highest coefficient of determination (R^2^), lowest Akaike information criterion (AIC), and highest model selection criterion (MSC) values. Model fitting was conducted using DD‐Solver software Version 1, a Microsoft Excel add‐in developed in Visual Basic for Applications.

### 2.6. Characterization of ISM Remnants

#### 2.6.1. Morphology

After the completion of the drug‐release test, the remaining ISM matrices were collected, freeze‐dried, and examined by scanning electron microscopy (SEM, JSM‐IT500HR, JEOL, Tokyo, Japan) after sputter‐coating with gold. SEM micrographs of intact GV, IBU, PA, and postrelease remnants were obtained at an accelerating voltage of 10 kV.

#### 2.6.2. Weight Loss and Water Tolerance

Weight loss was determined by immersing ISM matrices (0.5 g) in PBS (pH 6.8) at 37°C for 7 days. Samples were removed and dried to constant weight, and percentage weight loss was calculated (*n* = 3). Water tolerance was determined for GV‐free ISM formulations by titrating 2 mL of formulation with deionized water at 25°C until phase separation occurred. The maximum water tolerated before turbidity was recorded and reported at %water tolerance (*n* = 3).

### 2.7. Molecular Modeling Studies

Density functional theory (DFT) calculations were performed using Gaussian 16 software. The molecular structures of GV, IBU, DMSO, and NMP were optimized at the B3LYP‐D3BJ/6‐31g(d,p) level of theory. The inclusion of the D3BJ empirical dispersion correction was employed to adequately capture noncovalent interactions, including van der Waals and *π* − *π* stacking forces, which are particularly relevant for the large aromatic system of GV. Electrostatic potential (ESP) maps were generated for each compound. Formation energies of all possible binary configurations were calculated. The most stable 1:1 configuration was further analyzed, and ESP maps were plotted. It is acknowledged that the 6‐31G(d,p) basis set may be susceptible to basis set superposition error (BSSE) and that the calculated binding energies should be interpreted as semiquantitative estimates reflecting relative interaction trends among the formulation components rather than absolute values. BSSE correction via the counterpoise method and basis set refinement at a higher level of theory are recommended for future computational work to improve quantitative accuracy.

### 2.8. Antimicrobial Activity

The antimicrobial activity of GV‐loaded and control ISM formulations was determined using the agar diffusion method. Agar plates were inoculated with standardized microbial suspensions (10^6^ CFU/mL). Cylindrical wells (7 mm) were filled with 150 μL of ISM formulation, and plates were incubated at 37°C for bacteria (24 h) and 30°C for yeast (48 h). The diameter of inhibition zones was measured on Days 1, 7, and 15 (*n* = 3). The inhibition zone was defined as the clear, growth‐free halo surrounding the well, visually distinguished from the area of dye diffusion based on the presence or absence of visible microbial growth under direct illumination against a dark background. A 15‐day sustained antimicrobial profile was targeted in this study to provide coverage spanning the full treatment window while enabling a single application, thus potentially improving patient compliance and reducing the need for repeated dosing.

### 2.9. Statistical Analysis

All experiments were performed in triplicate (*n* = 3). The results are expressed as mean ± standard deviation (SD). Statistical analysis was performed using one‐way ANOVA, followed by Tukey’s post hoc test (SPSS Version 25.0, IBM Corp., Armonk, NY, USA). A *p* value of < 0.05 was considered statistically significant.

## 3. Results and Discussion

### 3.1. Physical Appearance and Formulation Composition

All GV‐loaded ISM formulations prepared using DMSO and NMP appeared as clear, purple, and homogeneous solutions, with no visible precipitation. This consistent clarity across various compositions confirms the successful solubilization of GV, IBU, and PA in both solvent systems. The binary and ternary combinations of IBU and PA, incorporated at concentrations ranging from 20% to 50% w/w, demonstrated favorable miscibility. These findings align with previous studies showing that low‐molecular‐weight hydrophobic drugs dissolve effectively in aprotic polar solvents like DMSO and NMP, forming injectable ISM systems [35].

### 3.2. Viscosity and Rheological Behavior

The viscosity of the formulations ranged from 5.77 to 23.38 cPs in DMSO‐based ISMs and 6.32 to 21.12 cPs in NMP‐based ISMs (Table 2). Increasing the concentration of IBU or PA led to a corresponding increase in viscosity. For example, in DMSO‐based systems, viscosity increased from 13.21 ± 0.23 cPs in GI40D to 18.79 ± 0.81 cPs in GI50D, from 15.17 ± 0.15 cPs in GP40D to 22.60 ± 2.96 cPs in GP50D, and from 13.66 ± 0.36 cPs in GI20P20D to 17.86 ± 0.59 cPs in GI25P25D. A similar pattern was observed in the NMP‐based ISMs. Interestingly, ternary formulations (e.g., GI20P20D and GI25P25D) containing both IBU and PA exhibited lower viscosities compared to their respective binary systems, despite similar total solute concentrations. For instance, GI25P25D exhibited a viscosity of 17.86 ± 0.59 cPs, which was lower than that of GI50D (18.79 ± 0.81 cPs) and GP50D (22.60 ± 2.96 cPs). This reduction may be attributed to a dilution effect, where each solute is present at a lower concentration, thus limiting the extent of solute–solvent interactions. Additionally, the coexistence of two structurally distinct solutes may disrupt molecular packing and hydrogen bonding networks, thereby mitigating steric hindrance and leading to lower viscosity in ternary systems [35–37].

**TABLE 2:** The viscosity, injectability, and mechanical properties of GV‐loaded ISMs and control formulations (*n* = 3).

Formulation	Viscosity (cPs)	Injectability		Mechanical properties			
		Force (N)	Energy (mJ)	Maximum force (N)	Adhesion (N)	Remaining force (N)	Remaining force/maximum force
GI40D	13.21 ± 0.23	0.96 ± 0.05	5.04 ± 0.16	—	—	—	—
GI50D	18.79 ± 0.81	1.09 ± 0.17	5.93 ± 0.12	—	—	—	—
GP40D	15.17 ± 0.15	1.13 ± 0.09	5.92 ± 0.22	4.79 ± 0.25	0.12 ± 0.01	0.70 ± 0.17	0.15 ± 0.03
GP50D	22.60 ± 2.96	1.14 ± 0.18	5.94 ± 0.04	5.37 ± 0.09	0.12 ± 0.04	1.66 ± 0.25	0.31 ± 0.05
GI20P20D	13.66 ± 0.36	1.07 ± 0.11	4.83 ± 0.21	1.28 ± 0.13	0.11 ± 0.01	0.29 ± 0.06	0.23 ± 0.05
GI25P25D	17.86 ± 0.59	0.99 ± 0.05	4.94 ± 0.23	4.06 ± 0.09	0.12 ± 0.02	0.90 ± 0.09	0.22 ± 0.03
I50D	17.48 ± 0.28	1.11 ± 0.09	5.58 ± 0.19	—	—	—	—
P50D	23.38 ± 2.47	1.29 ± 0.16	7.02 ± 0.71	15.92 ± 1.6	0.34 ± 0.13	7.35 ± 0.68	0.46 ± 0.04
GD	5.77 ± 0.47	1.05 ± 0.08	4.77 ± 0.22	—	—	—	—
DMSO	6.20 ± 0.50	—	—	—	—	—	—
GI40N	14.15 ± 0.31	1.09 ± 0.20	6.09 ± 0.09	—	—	—	—
GI50N	21.10 ± 0.33	0.97 ± 0.24	4.98 ± 0.24	—	—	—	—
GP40N	11.68 ± 0.20	1.24 ± 0.11	5.77 ± 0.43	3.22 ± 0.24	0.21 ± 0.04	1.10 ± 0.17	0.35 ± 0.08
GP50N	21.10 ± 0.33	1.25 ± 0.04	5.99 ± 0.38	4.36 ± 0.37	0.3 ± 0.08	3.06 ± 0.24	0.70 ± 0.02
GI20P20N	12.10 ± 0.27	0.95 ± 0.05	5.95 ± 0.44	1.27 ± 0.11	0.10 ± 0.01	0.56 ± 0.05	0.44 ± 0.01
GI25P25N	16.49 ± 0.26	0.78 ± 0.01	5.61 ± 0.53	1.80 ± 0.11	0.09 ± 0.01	0.69 ± 0.07	0.38 ± 0.03
I50N	21.12 ± 0.42	1.08 ± 0.09	6.68 ± 0.09	—	—	—	—
P50N	14.19 ± 1.29	1.50 ± 0.09	7.57 ± 0.95	4.80 ± 0.34	0.66 ± 0.04	3.20 ± 0.07	0.67 ± 0.04
GN	6.32 ± 0.72	0.91 ± 0.14	3.66 ± 0.24	—	—	—	—
NMP	7.78 ± 0.57	—	—	—	—	—	—

*Note:* (—) = not available.

**FIGURE 2:**
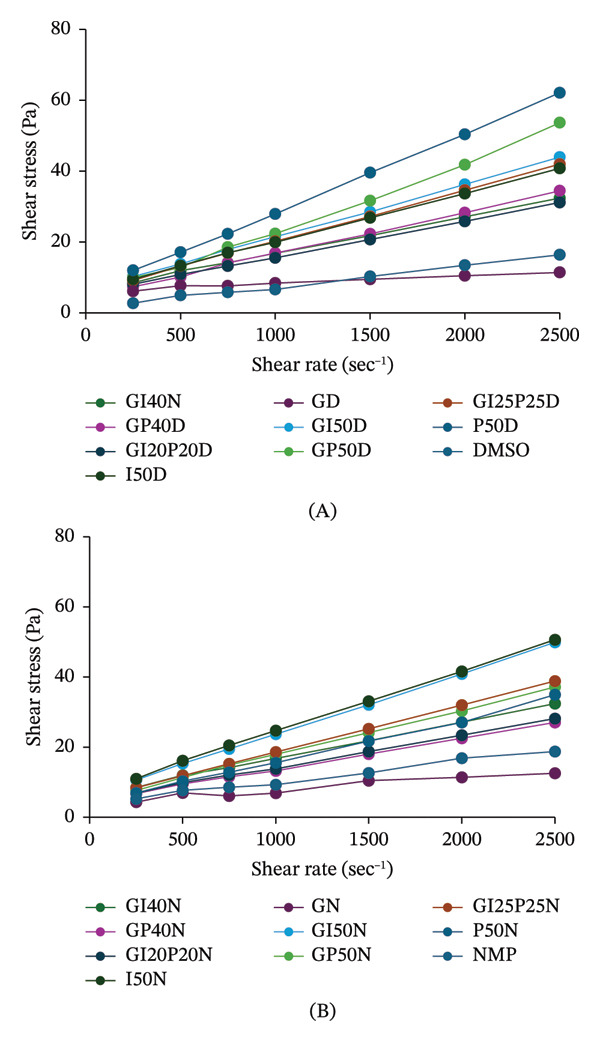
Rheological behavior of GV‐loaded ISMs and control formulations in DMSO (A) and NMP (B).

Figure 2 illustrates the shear stress–shear rate relationships of the GV‐loaded formulations. Across all tested samples, including those with higher concentrations of IBU and/or PA (e.g., GI50D, GP50D, GI25P25D, GI50N, GP50N, and GI25P25N), the curves exhibited a predominantly linear trend, indicating Newtonian‐flow behavior. This suggests that the viscosity of these systems remained relatively constant over the applied shear rate range, which is favorable for consistent injectability [38].

The data also show that a higher solute content (IBU and PA) increased the formulation viscosity. These viscosity increases are likely attributable to both the higher solute loading and potential intermolecular interactions. As the concentration of IBU and PA increases, the number of hydrophobic and hydrogen bonding interactions within the solution rises, such as hydrogen bonding between the carboxyl group of IBU and the sulfoxide in DMSO or lactam in NMP groups. The addition of PA, a long‐chain fatty acid, may also contribute to increased steric hindrance, thereby restricting solvent mobility and elevating viscosity. These effects collectively raise the apparent viscosity of the formulations. Although viscosity increased with solute loading, the rheological profiling (Figure 2) confirmed that the formulations composed of IBU and PA exhibited a linear shear stress–shear rate relationship, indicating Newtonian‐flow behavior. This contrasts slightly with a previous report, where higher concentrations of matrix‐forming agents led to shear‐thinning [39]. The differences highlight the influence of solute identity, solvent type, and molecular interactions in determining the flow characteristics of solvent exchange–induced ISMs. Furthermore, the formulations with Newtonian behavior suggested that the internal structure of the system remains relatively uniform and does not undergo shear‐induced structural rearrangement, which is favorable for predictable and consistent injectability in clinical applications for use via subgingival injection into periodontal pockets [27, 40, 41].

### 3.3. Injectability and Mechanical Properties

Injectability is a critical parameter for ISM systems intended for subgingival administration, as it directly influences clinical usability and patient comfort. The force required to expel the formulations through a standard syringe ranged from 0.78 to 1.50 N, indicating generally favorable injectability across all tested systems (Table 2). However, the relationship between viscosity and injection force was not strictly linear. For instance, P50D, which exhibited the highest viscosity (23.38 ± 2.47 cPs), required a relatively high injection force (1.29 ± 0.16 N), consistent with previous findings where an increased polymer content led to greater resistance during injection [42]. In contrast, GI25P25N, a ternary formulation with moderate viscosity (16.49 ± 0.26 cPs), demonstrated the lowest injection force (0.78 ± 0.01 N), suggesting that solute composition and molecular interactions may facilitate smoother flow. This observation aligns with a previous report where formulations containing IBU and doxycycline exhibited reduced injection resistance due to favorable solute–solvent dynamics and lower structural entanglement [43]. Additionally, formulations such as GN and GD, despite their low viscosities (6.32 ± 0.72 cPs and 5.77 ± 0.47 cPs, respectively), did not consistently show the lowest injection forces, indicating that injectability is influenced by more than viscosity alone, potentially including factors such as solute crystallinity, solvent polarity, and phase transition behavior. However, all formulations remained within the acceptable force limit for subgingival injection (< 50 N mm), supporting their suitability for periodontal pocket administration [44, 45].

Mechanical properties, including maximum force, adhesion, and remaining force, were determined for selected formulations to evaluate matrix robustness postinjection. Among them, P50D, with the highest mechanical strength, exhibited a maximum force of 15.92 ± 1.60 N and remaining force of 7.35 ± 0.68 N, resulting in a relatively high retention ratio (0.46 ± 0.04). These results suggest that the formulation forms a mechanically stable matrix upon solvent exchange, capable of withstanding deformation and resisting detachment under physiological conditions. This behavior is likely attributable to the high PA content, which enhances hydrophobic interactions and matrix integrity, as similarly observed in cellulosic ester‐based ISM systems with strong resistance to deformation and detachment, likely due to the high content of PA contributing to enhanced hydrophobic interactions and matrix cohesiveness [46].

Binary formulations such as GP50N and GP40N also showed enhanced mechanical performance compared to ternary systems. GP50N, for example, presented a maximum force of 4.36 ± 0.37 N and remaining force of 3.06 ± 0.24 N, resulting in a high retention ratio of 0.70 ± 0.02, the highest among NMP‐based formulations. This finding underscores the dominant role of PA in promoting matrix strength and resistance to removal. In contrast, ternary formulations such as GI20P20D, GI25P25D, GI20P20N, and GI25P25N, which contained lower total concentrations of IBU and PA (20%–25% w/w each), demonstrated moderate mechanical strength and lower retention ratios, ranging from 0.23 ± 0.03 to 0.44 ± 0.01, reflecting their reduced PA or IBU content. These values reflect the diminished cohesive and adhesive strength of formulations with lower matrix‐former content, which may impair their ability to withstand displacement by gingival crevicular fluid (GCF) or mechanical agitation within the periodontal pocket. The retention ratio, calculated as the ratio of remaining force to maximum force, reflects the elastic–plastic behavior of ISMs. While higher ratios indicate greater elasticity and resistance to dislodgement, excessive elasticity may reduce conformability. Conversely, lower ratios suggest plastic deformation, which enhances adaptation to periodontal pocket geometry but may compromise mechanical stability. Therefore, an optimal retention ratio balances elasticity for retention and plasticity for conformability [39, 47].

Comparing the solvent systems, DMSO‐based ISMs generally displayed higher viscosities and slightly greater mechanical retention than their NMP counterparts at equivalent solute compositions. This may be due to the higher intrinsic viscosity and polarity of DMSO, as well as its stronger hydrogen‐bonding capacity with functional groups in IBU and PA. Nonetheless, both solvents allowed the preparation of ISMs with acceptable injectability and rheological properties, underscoring their versatility in solubilizing small‐molecule hydrophobic agents.

### 3.4. *In Situ* Matrix‐Forming Behavior

**FIGURE 3:**
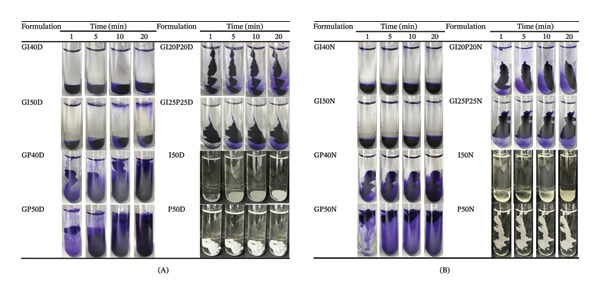
Matrix formation of ISM and control formulations dissolved in DMSO (A) and NMP (B) in PBS pH 6.8.

As shown in Figure 3A, both GV‐loaded and control formulations dissolved in DMSO underwent a clear sol‐to‐matrix transition upon contact with PBS pH 6.8. Initially, the injected formulations remained in a liquid state and settled at the bottom of the test tube. Within minutes, they transformed into translucent gel masses, indicating phase separation driven by solvent exchange. The GV‐loaded formulation exhibited a slightly delayed gelation compared to the control, possibly due to increased viscosity or molecular interactions involving GV that hindered solvent diffusion.

Among the formulations tested, those with high proportions of a single component (such as GI40D and GI50D and GP40D and GP50D) exhibited distinct gelation behaviors. GI40D and GI50D produced dense and opaque matrices, suggesting rapid phase separation. However, their high viscosity may impede syringeability, posing challenges for in vivo administration. In contrast, GP40D and GP50D formed softer gels. The higher lipid content appeared to slow down gel densification, resulting in matrices that were less compact, possibly by reducing internal phase separation forces or slowing solvent exchange. Interestingly, formulations with balanced ratios of IBU and PA, such as GI20P20D and GI25P25D, exhibited moderate and more controlled behavior. These formulations formed distinct but not overly rigid matrices, suggesting that optimal component ratios contribute to desirable matrix consistency and transformation rates.

When compared to NMP‐based formulations (Figure 3B), the gelation varied depending on the formulation composition. In general, DMSO‐based formulations showed more cohesive and structured matrix formation, particularly for GI40D and GI50D, which formed dense, opaque masses. In contrast, their NMP counterparts, GI40N and GI50N, appeared less structured and more dispersed, suggesting slower or incomplete gelation. However, GP40N and GP50N formed turbid but more evenly dispersed gel‐like masses, while the corresponding DMSO‐based formulations (GP40D and GP50D) produced weaker or less clearly defined gel masses. Similarly, GI20P20N and GI25P25N formed visible gels with gradual phase separation, comparable to or better than their DMSO counterparts.

These observations suggest that while DMSO tends to promote faster gelation in IBU‐rich formulations due to its higher polarity and miscibility with water, this effect can be diminished or altered in the presence of a higher PA content. Therefore, overall gelation performance is not solely dependent on the solvent type but also significantly influenced by the formulation composition. Both solvent properties and the drug–lipid ratio must be considered when optimizing ISM systems for balanced performance, injectability, and matrix structure.

**FIGURE 4:**
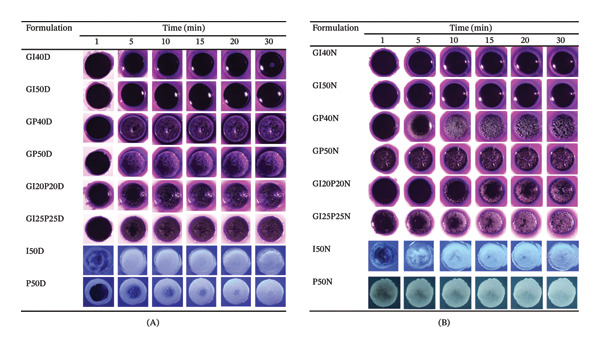
Matrix formation of ISM and control formulations dissolved in DMSO (A) and NMP (B) in an agarose well under a stereomicroscope at × 20 magnification.

Matrix formation was further investigated by stereomicroscopic imaging in agarose wells. The transformation from liquid to gel matrix was evident through the development of opaque rings and mass formation, indicative of phase inversion via solvent exchange. As shown in Figure 4, stereomicroscopic observation revealed notable differences in matrix formation among the tested formulations. For the DMSO‐based IBU‐rich formulations, GI40D and GI50D, no clear opaque zones or visible matrix expansion was observed within the agarose wells (Figure 4A). Unlike other formulations, these samples did not display gel spreading or the characteristic signs of phase separation typically seen during in situ transformation. This contrasts with prior findings, where DMSO‐based ISMs with high IBU content showed distinct opaque ring formation [43]. The discrepancy may be attributed to the presence of GV, a cationic aromatic compound that could interact with IBU and other formulation components. Such interactions may alter solvent exchange kinetics, increase viscosity, or stabilize IBU in solution, thereby suppressing phase separation and delaying matrix network formation.

Additionally, the intense violet coloration of GV may reduce optical contrast under stereomicroscopy, potentially obscuring weak or diffuse matrix structures. While this effect is less likely to fully account for the absence of visible gelation, it may contribute to the difficulty in detecting early phase inversion.

In contrast, the GP40D and GP50D formulations, containing a higher PA content, exhibited more evident matrix formation, characterized by soft, diffused opacity extending modestly into the agarose well. Although not highly dense, the visible spread suggests that PA may facilitate more gradual solvent diffusion and matrix network formation. Notably, GV did not appear to interfere with matrix formation in PA‐rich systems, possibly due to the absence of aromatic or acidic functional groups in PA that could interact with GV. This chemical inertness may allow solvent exchange and matrix formation to proceed more readily.

The ternary formulations, GI20P20D and GI25P25D, showed the most well‐defined matrix expansion among the DMSO series. These samples produced visible opaque rings with moderate matrix spread and relatively uniform edges. The findings indicate that balanced IBU–PA ratios promote controlled and efficient matrix transformation, likely due to an optimized interplay between hydrophobicity, viscosity, and solvent exchange dynamics.

Compared to their NMP‐based counterparts (Figure 4B), GI40N and GI50N also lacked prominent opaque ring formation. Interestingly, GP40N and GP50N formed soft, turbid matrices that were more uniformly dispersed than their DMSO counterparts. Similarly, GI20P20N and GI25P25N exhibited more cohesive matrix expansion with noticeably greater white opacity. These visual differences highlight the influence of solvent type and formulation composition on the matrix morphology and dispersion patterns within the agarose well.

### 3.5. *In Vitro* Drug Release

**FIGURE 5:**
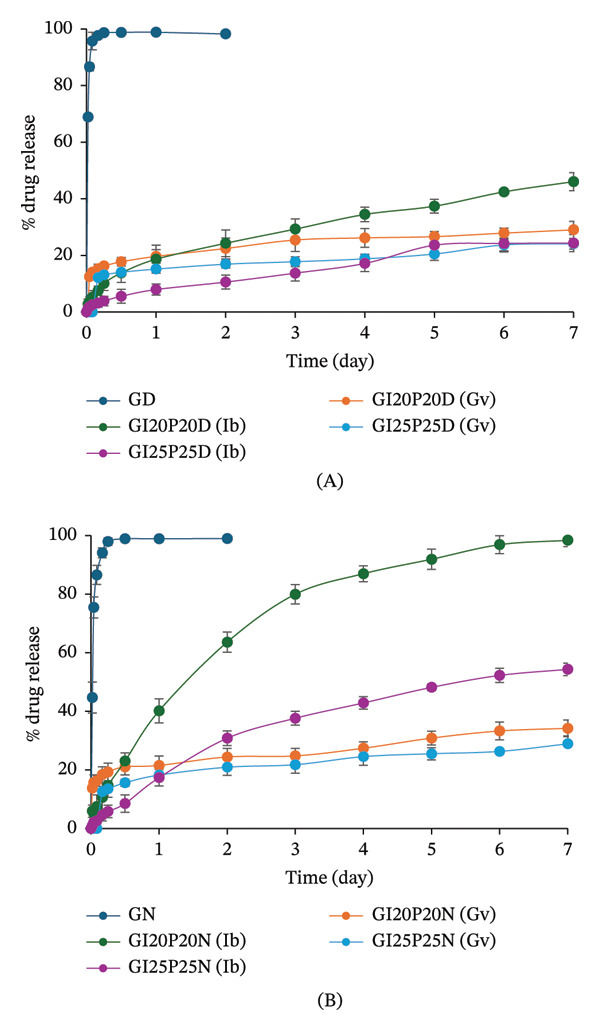
The release profile of GV and IBU (Ib) from GI20P20D and GI25P25D formulations (A) and from GI20P20N and GI25P25N formulations (B) (*n* = 3).

GV‐loaded ISM formulations were designed as localized drug delivery systems for periodontitis treatment, employing IBU and PA as dual‐function matrix formers. This strategy not only leveraged the anti‐inflammatory properties of IBU but also enabled sustained drug delivery through in situ gelation, in which GV was molecularly dispersed within the homogeneous solution of IBU and PA dissolved in either DMSO or NMP. Upon injection into an aqueous environment, solvent exchange triggered phase inversion, leading to matrix formation that physically entrapped GV within the solidified matrix network. This mechanism ensures localized, sustained drug release at the site of administration, with the matrix‐forming agents playing a dual role in both therapeutic action and drug entrapment. The in vitro drug‐release profiles of GV and IBU from the developed ISM formulations are presented in Figure 5, with kinetic parameters summarized in Tables 3 and 4. All formulations exhibited biphasic release patterns, characterized by an initial burst phase followed by a sustained‐release phase. The extent and rate of GV release were influenced by both the solvent type, DMSO or NMP, and polymer concentration (20% or 25%).

**TABLE 3:** Estimated parameters from the GV release profiles.

Formulation	Modeling	Criteria for model selection			Kinetic parameters	
		*R* ^2^	AIC	MSC		
GI20P20D	Zero order	—	—	—	—	
	First order	—	—	—	—	
	Higuchi’s	0.2776	38.3689	−1.3187	kH = 5.257	
	Korsmeyer–Peppas	0.6526	35.2445	−0.8723	kKP = 9.380	*n* = 0.261
	Peppas–Sahlin	0.7380	35.2692	−0.8758	*k*1 = 9.890 *k*2 = −1.234	*m* = 0.495

GI25P25D	Zero order	0.4203	38.6237	−0.5677	*k*0 = 0.849	
	First order	0.4696	38.0015	−0.4789	*k*1 = 0.010	
	Higuchi’s	0.6992	34.0311	0.0883	kH = 3.623	
	Korsmeyer–Peppas	0.6993	36.0299	−0.1972	kKP = 3.577	*n* = 0.505
	Peppas–Sahlin	0.8284	34.1048	0.0778	*k*1 = 2.430 *k*2 = −0.084	*m* = 0.949

GI20P20N	Zero order	—	—	—	—	
	First order	—	—	—	—	
	Higuchi’s	—	—	—	—	
	Korsmeyer–Peppas	0.9858	6.3679	2.9966	kKP = 15.415	*n* = 0.115
	Peppas–Sahlin	0.9881	2.3187	3.5750	*k*1 = 19.653 *k*2 = −4.358	*m* = 0.207

GI25P25N	Zero order	0.5528	38.3639	−0.2672	*k*0 = 0.973	
	First order	0.6034	37.5230	−0.1471	*k*1 = 0.011	
	Higuchi’s	0.7642	33.8843	0.3727	kH = 4.071	
	Korsmeyer–Peppas	0.76689	35.7435	0.1071	kKP = 3.596	*n* = 0.549
	Peppas–Sahlin	0.8626	34.1055	0.3411	*k*1 = 2.424 *k*2 = −0.074	*m* = 0.968

**TABLE 4:** Estimated parameters from the IBU (Ib) release profiles.

Formulation	Modeling	Criteria for model selection			Kinetic parameters	
		*R* ^2^	AIC	MSC		
GI20P20D	Zero order	0.6115	32.5341	0.6597	*k*0 = 0.925	
	First order	0.6757	31.2695	0.8403	*k*1 = 0.010	
	Higuchi’s	0.9956	1.1284	5.1462	kH = 3.873	
	Korsmeyer–Peppas	0.9956	3.1205	4.8616	kKP = 3.886	*n* = 0.499
	Peppas–Sahlin	0.9978	0.4236	5.2469	*k*1 = 3.776 *k*2 = −0.144	*m* = 0.596

GI25P25D	Zero order	0.5774	20.3269	0.5755	*k*0 = 0.386	
	First order	0.6032	19.8854	0.6386	*k*1 = 0.004	
	Higuchi’s	0.9964	−13.0441	5.3428	kH = 1.618	
	Korsmeyer–Peppas	0.9971	−12.5935	5.2785	kKP = 1.682	*n* = 0.484
	Peppas–Sahlin	0.9991	−18.3899	6.1065	*k*1 = 1.351 *k*2 = 0.388	*m* = 0.356

GI20P20N	Zero order	0.9064	33.4350	2.0831	*k*0 = 1.793	
	First order	0.9454	29.6629	2.6220	*k*1 = 0.023	
	Higuchi’s	0.9339	31.0008	2.4309	kH = 7.141	
	Korsmeyer–Peppas	0.9849	22.6678	3.6213	kKP = 4.417	*n* = 0.687
	Peppas–Sahlin	0.9906	21.3118	3.8150	*k*1 = 3.939 *k*2 = 0.916	*m* = 0.492

GI25P25N	Zero order	0.9523	17.3074	2.7581	*k*0 = 0.746	
	First order	0.9608	15.9422	2.9531	*k*1 = 0.008	
	Higuchi’s	0.8994	22.5363	2.920	kH = 2.920	
	Korsmeyer–Peppas	0.9846	11.4026	3.6016	kKP = 1.439	*n* = 0.773
	Peppas–Sahlin	0.9900	10.4054	3.7441	*k*1 = 1.417 *k*2 = 0.210	*m* = 0.576

GV demonstrated a slower release profile than IBU across all formulations, which can be attributed to its higher molecular weight and lower aqueous solubility. Compared to their NMP‐based counterparts, the DMSO‐based formulations, GI20P20D and GI25P25D, exhibited slower GV release, with 29.02% and 24.08% of drug released by Day 7, respectively. The initial lag phase observed in GI25P25D suggests delayed gelation due to the higher polymer content, which likely promoted a denser matrix with reduced porosity, impeding solvent penetration and GV diffusion. The release data of GI25P25D best fit the Peppas–Sahlin model (*R* ^2^ = 0.8284, AIC = 34.10), with a diffusional exponent *m* = 0.949, indicating anomalous transport involving both diffusion and matrix relaxation. In contrast, GI20P20D showed a more diffusion‐dominant release (*m* = 0.495, *n* = 0.261), reflecting Fickian behavior through a less compact matrix. These findings are consistent with previous studies demonstrating that DMSO promotes rapid phase inversion and formation of tightly packed polymeric networks that slow down drug diffusion [43].

In comparison, the NMP‐based formulations, GI20P20N and GI25P25N, demonstrated enhanced GV release, with cumulative values of 34.20% and 28.89% by Day 7, respectively. This higher release rate is attributable to the slower solvent exchange of NMP, which allows extended molecular rearrangement during gelation and results in more porous, less rigid matrices. As reported by Puyathorn et al. [43], NMP‐based ISMs tend to form softer structures that permit greater water influx and drug mobility. GI20P20N exhibited a rapid burst release, best described by the Peppas–Sahlin model (*R* ^2^ = 0.9881), with a low diffusional exponent (*m* = 0.207, *n* = 0.115), confirming a Fickian diffusion mechanism. The GI25P25N formulation, with higher polymer content, showed a more gradual release, transitioning toward anomalous transport (*n* = 0.549), where both diffusion and polymer relaxation influence release kinetics.

**FIGURE 6:**
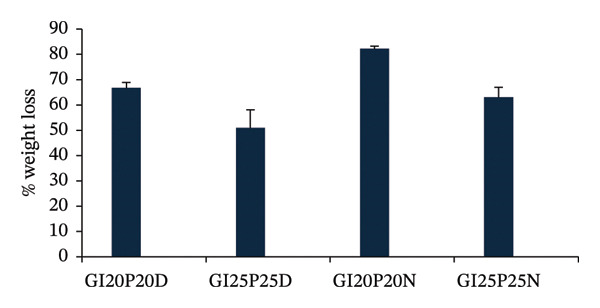
Weight loss value of GV‐loaded ISM formulations after drug‐release test (*n* = 3).

The release study findings were further supported by the weight loss analysis of dried matrix remnants after drug release (Figure 6). NMP‐based formulations exhibited greater weight reduction compared with their DMSO counterparts, with GI20P20N showing more than 80% weight loss versus approximately 67% for GI20P20D. This difference suggests that the slower solvent exchange of NMP produced more porous, sponge‐like matrices that facilitated faster drug release and higher structural erosion. In contrast, the denser matrices formed in DMSO resulted in slower drug diffusion and lower weight loss. Increasing the concentrations of IBU and PA reduced the overall weight loss in both solvent systems, indicating that higher polymer content enhanced matrix compactness, limited water penetration, and consequently slowed both drug release and matrix degradation.

The release behavior of IBU followed similar trends but generally proceeded faster than GV due to its lower molecular weight and higher water solubility. IBU release from GI20P20D and GI25P25D closely followed the Higuchi model (*R* ^2^ > 0.995), suggesting a dominant diffusion‐controlled mechanism through the solid matrix. These results were further supported by excellent fits to the Peppas–Sahlin model (*R* ^2^ > 0.9978), with *m* values < 0.6, confirming quasi‐Fickian diffusion. Similarly, IBU release from GI20P20N and GI25P25N demonstrated high cumulative release and strong model fitting, with GI25P25N exhibiting slightly more sustained release due to its denser matrix (*R* ^2^ = 0.9900, *m* = 0.576).

The inclusion of PA as a comatrix former likely contributed to these release patterns. Its crystalline nature and low molecular weight promote rapid precipitation during solvent exchange, enhancing matrix porosity and thereby modulating drug diffusion. Previous reports on CAB/PA‐based ISMs have highlighted similar structural effects of PA in creating more permeable matrices [27]. In this study, its contribution was particularly evident in GI25P25 formulations, where the balance between matrix compactness and porosity supported sustained release.

These observations are consistent with earlier findings from DH‐loaded IBU‐based ISMs, which also demonstrated biphasic release characterized by an initial burst followed by a slower sustained phase due to matrix solidification [48]. Previous studies have also highlighted the importance of solvent selection and polymer architecture in controlling drug diffusion and maintaining matrix integrity in ISM systems [49].

Altogether, the GV and IBU release profiles from the current ISM formulations confirm that solvent type, polymer concentration, and matrix‐forming agents synergistically influence drug delivery behavior. The Peppas–Sahlin model provided the best overall fit, reflecting the combined effects of diffusion and structural relaxation. These ISM systems demonstrated sustained release for over 7 days, highlighting their potential as effective, locally applied therapies for periodontitis. The dual‐drug, dual‐function approach further supports improved patient compliance and therapeutic efficacy by integrating anti‐inflammatory and antimicrobial activities within a single injectable system.

### 3.6. Water Tolerance

**FIGURE 7:**
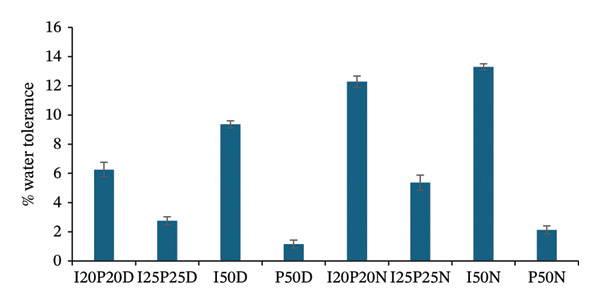
Water tolerance value of ISM formulations without GV after titration with deionized water (*n* = 3).

The results of the water tolerance test (Figure 7) revealed that NMP‐based ISM formulations exhibited higher tolerance values than those prepared with DMSO, indicating slower solvent exchange and delayed phase inversion. This observation is consistent with previous reports showing that DMSO‐based ISMs undergo more rapid phase inversion due to stronger solvent–nonsolvent interactions [50–52]. Increasing the total concentrations of IBU and PA led to a decrease in water tolerance, reflecting the enhanced hydrophobicity and reduced miscibility of the formulations. At higher IBU concentrations, phase separation occurred more readily upon water contact as IBU solubility in the solvent decreased [53]. The inclusion of the hydrophobic PA further reduced water tolerance by increasing the hydrophobic volume within the system and promoting structural transitions from water‐in‐oil emulsions to more ordered reverse hexagonal arrangements, as previously reported [54]. Among the single‐component systems, IBU‐based formulations showed greater water tolerance than PA‐based ones, likely due to the higher rigidity and lower flexibility of PA matrices, which limit water diffusion and phase adjustment in aqueous environments [55].

### 3.7. Surface Topography

**FIGURE 8:**
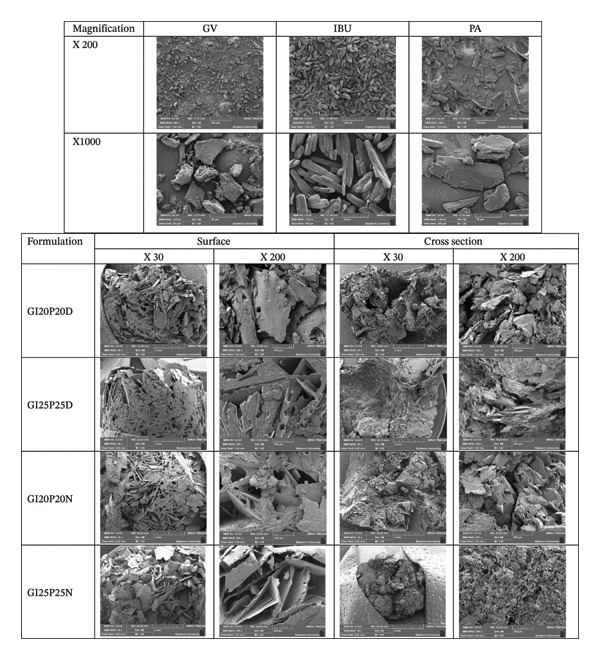
SEM micrograph of intact GV, IBU and PA (A); remnants of GV‐loaded ISMs after drug‐release test for 7 days.

The surface and cross‐sectional morphologies of GV‐loaded ISM formulations were characterized by scanning electron microscopy (SEM), as presented in Figure 8. The SEM micrographs revealed distinct morphological features among the various formulations, reflecting the influence of formulation composition and solvent systems on the matrix architecture.

SEM images of the intact substances, GV, IBU, and PA, showed inherent crystalline characteristics with varying shapes and surface textures. GV appeared as irregularly shaped particles with rough surfaces, while IBU displayed smoother, rhombic prism‐like crystals. PA was observed as compact, needle‐like structures. These inherent differences are important, as they can influence the initial dispersion, phase behavior, and recrystallization tendencies within the ISM matrix during solvent‐induced transformation.

Postdrug‐release SEM images of the ISM remnants demonstrated substantial morphological changes indicative of phase transformation and solvent exchange dynamics. The DMSO‐based formulations, GI20P20D and GI25P25D, displayed relatively compact, layered sheet‐like matrices with low porosity. These structures suggest a rapid solvent exchange process, promoting fast precipitation of matrix components and limiting the time available for molecular rearrangement. The observed compactness and denser matrix of GI25P25D may be attributed to the higher content of IBU and PA, which likely increased matrix rigidity. Such structural features are consistent with more controlled and sustained drug‐release behavior, as diffusion is limited by reduced pore interconnectivity and tortuosity.

In contrast, NMP‐based formulations (GI20P20N and GI25P25N) exhibited more porous and loosely packed surface morphologies, with increased opacity and a less compact network. The slower solvent exchange rate of NMP likely allowed extended molecular rearrangement and partial recrystallization, leading to a more open and heterogeneous structure. These topographical characteristics, particularly evident in GI25P25N, may contribute to enhanced water ingress and faster drug diffusion, supporting a biphasic release profile with an initial burst phase followed by sustained release. This structural difference aligns with prior studies, where slower phase inversion from NMP led to looser matrix packing and higher porosity in ISM systems [27].

Cross‐sectional SEM analysis further supported these findings. DMSO‐based formulations showed a gradient in matrix density from the surface to the interior, reflecting rapid surface precipitation followed by slower internal gelation. In contrast, the NMP‐based matrices demonstrated a more uniform, sponge‐like porous structure throughout the matrix, indicating a more homogeneous and gradual phase transformation process [56].

### 3.8. Molecular Modeling Studies

**FIGURE 9:**
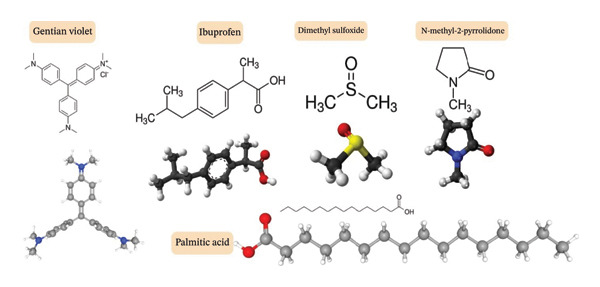
Optimized structures of GV, IBU, DMSO, and NMP with their electrostatic potential maps, calculated at the B3LYP‐D3BJ/6‐31G(d,p) level. Hydrogen, carbon, oxygen, sulfur, and nitrogen are represented in white, gray, red, yellow, and blue, respectively.

**FIGURE 10:**
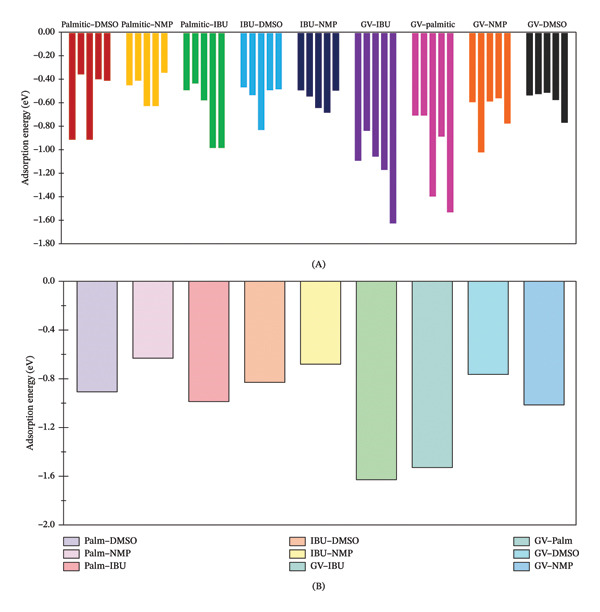
(A) Formation energy of all configurations and (B) the most stable configurations calculated at the B3LYP/6‐31G(d,p) level.

The optimized 3D structures of GV, IBU, PA, DMSO, and NMP are presented in Figure 9. The adsorption energy results obtained from DFT calculations at the B3LYP/6‐31G(d,p) level (Figure 10) revealed distinct interaction strengths among the components of the ISM systems. Generally, more negative adsorption energy values indicate stronger molecular interactions and greater thermodynamic stability of the complexes. The formation energies of all possible configurations are shown in Figure 10A, while the most stable configurations are depicted in Figure 10B.

**FIGURE 11:**
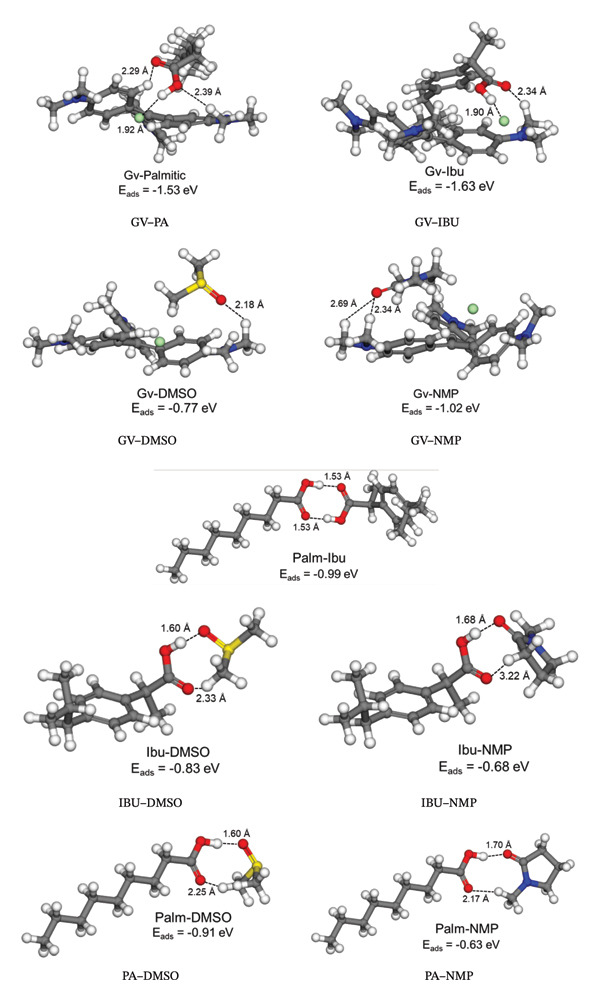
Optimized 1:1 configuration with electrostatic potential (ESP) maps showing intermolecular interactions between ibuprofen (IBU), palmitic acid (palm), gentian violet (GV), and solvents (DMSO or NMP), calculated at the B3LYP/6‐31G(d,p) level.

PA exhibited relatively weak interactions with both solvents, with adsorption energies of approximately −0.9 eV for palm–DMSO and −0.6 eV for palm–NMP, indicating limited polarity‐driven association due to its hydrophobic nature [57, 58]. In contrast, the palm–IBU pair predicted slightly stronger binding (≈−1.0 eV), suggesting favorable van der Waals and hydrogen‐bonding interactions between their carboxylic acid groups, as visualized in the ESP maps (Figure 11).

IBU showed stronger interactions with DMSO (≈−0.9 eV) compared to NMP (≈−0.7 eV), consistent with DMSO’s higher polarity and hydrogen‐bonding capability [59]. These stronger IBU–DMSO interactions likely contribute to the faster solvent exchange and denser matrix formation observed experimentally in DMSO‐based ISMs.

Among all investigated complexes, GV exhibited the most pronounced interaction energies, ranging from approximately −1.3 to −1.7 eV. The strongest binding occurred with GV–PA and GV–IBU, followed by GV–DMSO and GV–NMP. The strong GV–PA and GV–IBU interactions suggest that GV forms stable associations within the hydrophobic domains of the matrix, contributing to its sustained drug‐release behavior. This observation aligns with previous findings indicating that van der Waals dispersion forces and π–π stacking between aromatic rings are key noncovalent interactions stabilizing large aromatic molecules like GV [60]. The slightly higher stabilization of GV–DMSO compared to GV–NMP suggests that DMSO provides a stronger solvation environment, promoting uniform molecular dispersion prior to gelation. This enhanced stability likely arises from stronger hydrogen bonding between GV and the aprotic solvent, as evidenced by the ESP maps (Figure 11) and supported by similar reports describing hydrogen bonding and π–π interactions as dominant forces in GV binding [61]. While the DFT analysis revealed favorable interactions between GV and the matrix components, the potential for adverse ionic interactions arising from the cationic nature of GV and the carboxylic acid groups of IBU and PA warrants consideration as well. In the present study, no visible precipitation or phase separation was observed across all formulations, suggesting physical compatibility under the preparation conditions.

The ESP maps (Figure 11) illustrate the electron density distribution within the studied molecules, where electron‐rich regions (negative potential, red) are localized around carbonyl and oxygen atoms, while electron‐deficient regions (positive potential, blue) occur near hydrogens and selected nitrogen centers. These complementary charge patterns drive hydrogen bonding and electrostatic attraction among the components. Optimized geometries revealed several short intermolecular contacts (H···O or H···N distances 1.5–2.4 Å), consistent with moderate‐to‐strong polar interactions, for example, 1.60 and 2.33 Å for IBU–DMSO, 1.60 and 2.25 Å for PA–DMSO, 1.90 and 2.34 Å for GV–IBU, and 1.92–2.39 Å for GV–PA—while longer separations, such as the 3.22 Å observed for IBU–NMP, indicated weaker contacts.

The adsorption energy (*E*_ads_) results supported these findings. GV showed the strongest binding affinity, particularly with IBU (−1.63 eV) and PA (−1.53 eV), reflecting multicontact associations mediated by hydrogen bonding, van der Waals forces, and π–π stacking between GV’s aromatic rings and the carboxylic groups of IBU and PA. These strong interactions explain GV’s slower release, as it remains strongly retained within the hydrophobic domains of the matrix. The PA–IBU pair also exhibited notable stabilization (*E*_ads_ = −0.99 eV), indicating cooperative hydrogen bonding that promotes matrix integrity and controls porosity. The stronger GV–PA interaction compared with IBU–PA corresponds with the experimental release data, in which GV diffused more slowly than IBU in both DMSO‐ and NMP‐based systems, confirming tighter GV entrapment within the matrix phase. This finding is consistent with previous reports showing that lower formation energy results in more stable complexes, which in turn yield slower release of the active compound [62].

DMSO complexes were more stabilized than NMP counterparts (e.g., PA–DMSO = −0.91 eV vs. PA–NMP = −0.63 eV) due to DMSO’s strong hydrogen bond–accepting capacity [63]. This enhanced solvation promotes faster solvent exchange and the formation of dense, less porous matrices, consistent with the lower water tolerance and slower drug release of DMSO‐based formulations. In contrast, weaker NMP interactions result in slower solvent exchange, yielding more porous structures and faster release. This agrees with prior findings that rapid solvent exchange generates smoother, compact matrices, while slower exchange produces porous networks with faster drug diffusion [64]. GV–solvent interactions were weaker than GV–matrix interactions, although GV–NMP was slightly more stabilized than GV–DMSO, suggesting mild preferential affinity toward NMP.

Overall, the computational results closely align with experimental observations. Stronger GV–matrix binding and greater DMSO solvation correspond to slower release and lower water tolerance, whereas weaker NMP interactions support porous matrix formation and enhanced diffusion. These molecular‐level insights highlight the critical influence of solvent polarity, component compatibility, and intermolecular forces on the matrix structure and drug‐release behavior in the developed ISM systems. It is important to note that the molecular interactions discussed in this section are based on computational DFT analysis. Solid‐state characterization techniques such as FTIR spectroscopy and differential scanning calorimetry (DSC) were not performed in the present study. These analyses are recommended in future work to experimentally validate the predicted interaction modes and confirm physical compatibility between GV, IBU, PA, and the solvents in the developed ISM systems.

### 3.9. Antimicrobial Activities

The antimicrobial activities of GV‐loaded ISM formulations and control formulations against *S. aureus* ATCC 6538, *C. albicans* ATCC 10231, *C. tropicalis* TISTR 5306, and *P. gingivalis* ATCC 33277 were evaluated using the agar diffusion method over 15 days (Table 5).

**TABLE 5:** Antimicrobial activity of GV‐loaded ISM and control formulations in DMSO and NMP for Days 1, 7, and 15 against four microbes (*n* = 3).

Formulations	Inhibition zone diameter (mm. ± S.D.)			
	*S. aureus* 6538	*C. albicans* 10,231	*C. tropicalis* TISTR 5306	*P. gingivalis* 33,277
*DMSO-based ISM* Day 1				
GD	27.0 ± 1.0^A^	31.0 ± 1.0^A^	27.7 ± 1.2^A^	18.7 ± 0.6^A^
GI40D	21.7 ± 0.6^C,D^	26.0 ± 0.0^B,C,D^	24.0 ± 1.0^A,B,C,D^	15.7 ± 0.6^A,B,C,D,E^
GI50D	17.0 ± 1.0^F^	19.3 ± 0.6^E,F,G,H^	18.7 ± 0.6^E,F^	12.3 ± 0.6^F,G,H^
GP40D	25.3 ± 0.6^A,B^	29.0 ± 2.0^A,B^	25.7 ± 0.6^A,B^	18.3 ± 0.6^A^
GP50D	23.0 ± 0.0^B,C^	26.7 ± 2.3^A,B,C^	24.3 ± 1.2^A,B,C^	17.0 ± 0.0^A,B^
GI20P20D	19.7 ± 0.6^D,E,F^	21.3 ± 1.2^D,E,F,G^	19.7 ± 1.2^D,E,F^	12.7 ± 0.6^E,F,G,H^
GI25P25D	18.7 ± 0.6^E,F^	18.7 ± 1.5^F,G,H,I^	18.3 ± 0.6^E,F^	13.0 ± 1.0^D,E,F,G,H^
I50D	Nz	14.3 ± 0.6^I,J^	13.7 ± 0.6^G,H^	12.3 ± 0.6^F,G,H^
P50D	Nz	11.0 ± 1.0^J^	10.0 ± 0.0^H^	Nz
DMSO	11.3 ± 0.6^G^	16.3 ± 1.2^H,I^	16.0 ± 1.0^F,G^	10.0 ± 0.0^H^
Day 7				
GP40D	23.3 ± 0.6^B,C^	24.0 ± 1.0^C,D,E^	23.3 ± 0.6^A,B,C,D^	15.0 ± 0.0^B,C,D,E,F^
GP50D	23.7 ± 0.6^B,C^	21.3 ± 1.2^D,E,F,G^	21.7 ± 0.6^B,C,D,E^	15.0 ± 1.0^B,C,D,E,F^
GI20P20D	24.0 ± 1.0^B,C^	17.3 ± 2.5^G,H,I^	13.7 ± 2.5^G,H^	13.3 ± 0.6^C,D,E,F,G^
GI25P25D	18.3 ± 0.6^E,F^	17.0 ± 3.6^G,H,I^	18.0 ± 2.6^E,F,G^	11.3 ± 0.6^G,H^
Day 15				
GP40D	20.0 ± 1.0^D,E^	22.3 ± 0.6^C,D,E,F^	20.3 ± 0.6^C,D,E,F^	12.7 ± 0.6^E,F,G,H^
GP50D	22.0 ± 0.0^C,D^	22.7 ± 0.6^C,D,E,F^	19.7 ± 1.2^D,E,F^	12.3 ± 1.2^F,G,H^
GI20P20D	23.3 ± 1.5^B,C^	17.0 ± 1.0^G,H,I^	17.7 ± 2.5^E,F,G^	16.0 ± 1.0^A,B,C,D^
GI25P25D	18.0 ± 2.0^E,F^	16.3 ± 1.5^H,I^	17.0 ± 2.6^F,G^	16.3 ± 3.2^A,B,C^

*NMP-based ISM* Day 1				
GN	26.7 ± 0.6^a^	31.0 ± 1.0^a^	27.7 ± 0.6^a,b^	22.3 ± 0.6^a^
GI40N	25.3 ± 0.6^a,b^	33.3 ± 1.2^a^	29.7 ± 1.2^a^	22.0 ± 0.0^a^
GI50N	24.7 ± 0.6^a,b,c^	32.3 ± 1.5^a^	28.3 ± 1.2^a,b^	21.0 ± 0.0^a^
GP40N	22.7 ± 0.6^c,d^	23.7 ± 0.6^b^	23.7 ± 0.6^c,d^	16.3 ± 0.6^b,c^
GP50N	20.7 ± 0.6^d,e^	23.0 ± 1.0^b,c^	22.3 ± 0.6^c,d,e,f^	13.3 ± 0.6^e,f,g^
GI20P20N	22.0 ± 0.0^d^	30.3 ± 0.6^a^	25.3 ± 0.6^b,c^	17.3 ± 0.6^b^
GI25P25N	19.3 ± 0.6^e^	20.3 ± 2.1^b,c,d^	21.0 ± 0.0^d,e,f,g^	15.0 ± 0.0^c,d,e^
I50N	12.3 ± 1.2^f^	20.7 ± 1.5^b,c,d^	20.3 ± 0.6^e,f,g^	13.3 ± 0.6^e,f,g^
P50N	Nz	12.0 ± 0.0^f^	13.3 ± 1.2^h^	Nz
NMP	14.0 ± 1.0^f^	22.0 ± 2.0^b,c,d^	23.3 ± 0.6^c,d,e^	15.0 ± 0.0^c,d,e^
Day 7				
GP40N	23.0 ± 0.0^b,c,d^	24.0 ± 1.0^b^	22.0 ± 0.0^d,e,f,g^	14.7 ± 0.6^c,d,e^
GP50N	22.3 ± 0.6^c,d^	24.3 ± 2.1^b^	22.7 ± 0.6^c,d,e^	14.3 ± 0.6^d,e,f^
GI20P20N	21.3 ± 1.5^d,e^	18.7 ± 2.5^d^	15.7 ± 1.5^h^	14.7 ± 0.6^c,d,e^
GI25P25N	19.0 ± 1.0^e^	14.3 ± 0.6^e^	13.3 ± 2.1^h^	15.3 ± 0.6^c,d^
P50N	Nz	Nz	Nz	Nz
Day 15				
GP40N	23.0 ± 1.0^b,c,d^	23.0 ± 0.0^b,c^	19.0 ± 0.0^g^	12.7 ± 0.6^f,g^
GP50N	22.0 ± 1.0^d^	23.7 ± 0.6^b^	19.3 ± 1.2^f,g^	12.3 ± 0.6^g^
GI20P20N	26.3 ± 0.6^a^	19.0 ± 1.0^c,d^	19.0 ± 1.0^g^	14.3 ± 1.2^d,e,f^
GI25P25N	15.3 ± 0.6^a,b^	18.0 ± 1.0^d,e^	21.3 ± 1.5^d,e,f,g^	15.0 ± 1.0^c,d,e^

*Note:* nz = no inhibition zone; within solvent‐based, means that do not share a letter are significantly different.

**FIGURE 12:**
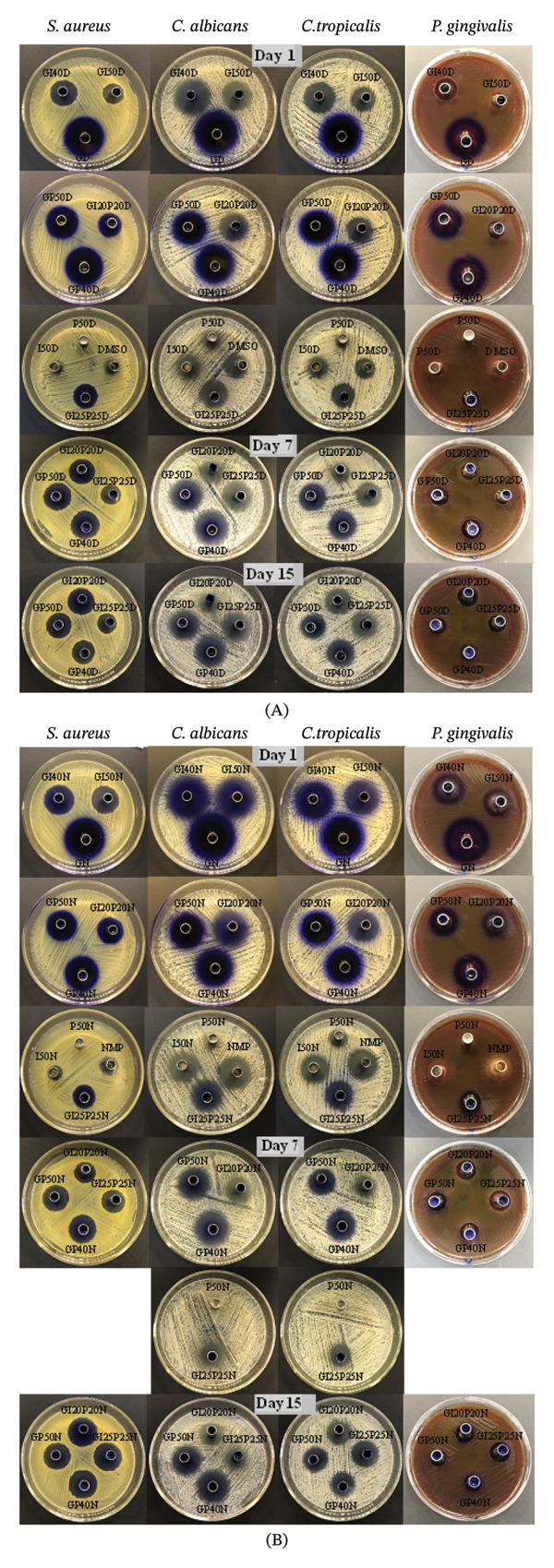
Photographs of the inhibition zone of GV‐loaded ISM and control formulations in DMSO (A) and NMP (B) for Days 1, 7, and 15 against four microbes.

Statistical analysis using one‐way ANOVA followed by Tukey’s post hoc test (*p* < 0.05) demonstrated significant differences among formulations and microbial strains. All formulations containing GV exhibited clear inhibition zones against both bacterial and fungal strains, confirming the preserved antimicrobial efficacy of GV after incorporation into the ISM systems (Figure 12). On Day 1, DMSO‐based GV formulations (GD, GP40D, and GP50D) and NMP‐based counterparts (GN, GI40N, and GI50N) demonstrated a strong activity, with inhibition zones exceeding 25 mm for *C. albicans* and *C. tropicalis* (*p* < 0.05), indicating potent antifungal effects consistent with GV’s known mechanism of disrupting fungal cell walls [65, 66]. Among all groups, NMP‐based formulations showed slightly higher inhibition zones than DMSO‐based systems, particularly against *P. gingivalis* (22.3 ± 0.6 mm for GN vs. 18.7 ± 0.6 mm for GD). This difference may partly arise from the intrinsic antimicrobial activity of NMP [67], which can disrupt microbial membranes and enhance drug efficacy [68]. Formulations containing both IBU and PA (GI20P20D/N and GI25P25D/N) exhibited slightly reduced inhibition zones compared with single‐matrix formulations, consistent with the denser matrix networks that retard early drug diffusion. However, these dual‐matrix systems maintained antimicrobial activity over prolonged periods, suggesting sustained GV release. For example, GI20P20D retained inhibition against all tested organisms up to Day 15 (*S. aureus* = 23.3 ± 1.5 mm; *P. gingivalis* = 16.0 ± 1.0 mm), while GI20P20N showed comparable long‐term activity (*S. aureus* = 26.3 ± 0.6 mm; *P. gingivalis* = 14.3 ± 1.2 mm) (*p* < 0.05).

The gradual reduction in the inhibition zone diameter over time was observed for all formulations, consistent with sustained‐release behavior. Notably, NMP‐based formulations preserved larger inhibition zones for *C. albicans* and *C. tropicalis* on Day 15 compared with DMSO‐based systems, indicating prolonged antifungal activity. This prolonged antifungal activity may be attributed to a synergistic effect between GV and NMP, as NMP itself exhibits antifungal properties [69]. Additionally, this observation aligns with the molecular modeling and release data, which demonstrated that weaker solvent–matrix interactions in NMP‐based systems promote the formation of more porous matrices, facilitating enhanced drug diffusion and sustained antimicrobial action. Control formulations containing only IBU or PA showed minimal inhibition against the tested microorganisms, confirming that the antimicrobial activity primarily originated from GV. However, the inclusion of IBU and PA appeared to modulate the matrix structure and release kinetics, indirectly influencing the diffusion and availability of GV at the microbial interface. Based on published data, the MIC range of GV for all Candida isolates tested was reported as 0.03–0.25 μg/mL [70]. The total GV content per formulation (0.4 g at 1% w/w = 4 mg GV) provides a substantial drug reservoir. Based on our cumulative release data, GI20P20N released approximately 34.2% of GV by Day 7, corresponding to an estimated cumulative release of ∼1.37 mg over 7 days. Given the small volume of a periodontal pocket (∼0.5–1 mL), the local GV concentrations achievable from these ISMs are expected to substantially exceed the MIC values reported for all tested pathogens throughout the treatment period. Overall, the results demonstrate that GV‐loaded ISM formulations effectively inhibit both bacterial and fungal pathogens relevant to OPC and periodontitis. The sustained antimicrobial activity over 15 days highlights the potential of these dual‐function, solvent‐induced in situ forming systems for localized infection control. NMP‐based ISMs offered slightly higher and longer‐lasting antimicrobial efficacy, correlating with their more open matrix structure and faster release profile. Despite GV has demonstrated an acceptable safety profile in short‐term clinical studies [6, 7], the 15‐day sustained‐release duration evaluated in this study warrants careful consideration regarding potential mucosal exposure. Under in vivo conditions, local concentrations would be further modulated by salivary dilution, GCF dynamics, and mucosal clearance mechanisms. Future studies should include cytotoxicity assessment using oral epithelial cell lines to confirm biocompatibility at the released concentrations. Similarly, while NMP has been widely employed as an ISM solvent in preclinical studies [21–23], its clinical acceptability for direct periodontal application requires further validation. In addition, although the agar diffusion method employed in this study provides a useful and widely accepted preliminary assessment of antimicrobial efficacy, it is recognized as a limitation for fully characterizing release‐dependent activity from an in situ forming depot system. In this method, drug diffusion from the liquid or partially solidified formulation into agar may not accurately replicate drug release from a fully formed matrix under physiological conditions. Future work should also employ time‐kill assays or a membrane‐based release diffusion model that better mimics the release kinetics from the solidified matrix to provide more clinically relevant antimicrobial data.

## 4. Conclusion

GV‐loaded ISMs using IBU and PA as dual‐function matrix formers were successfully developed for the localized treatment of OPC and periodontitis. DMSO‐based ISMs formed dense matrices with slower drug release, while NMP‐based systems generated more porous structures, enhancing diffusion. Release data fitted the Peppas–Sahlin model, indicating diffusion and matrix relaxation mechanisms. Molecular modeling computationally predicted strong GV–IBU and GV–PA interactions that stabilized the matrix and sustained release. All formulations exhibited potent, long‐lasting antimicrobial activity for up to 15 days, with NMP‐based systems showing slightly higher efficacy due to synergistic GV–NMP effects. These findings highlight GV‐loaded ISMs as promising localized delivery systems combining antimicrobial and anti‐inflammatory actions. However, several limitations of the present study should be acknowledged. Formal stability evaluation of the developed ISM formulations was not performed, and stability assessment is a critical requirement prior to any clinical or preclinical application. Safety and biocompatibility evaluation was also not conducted; the cytotoxicity of GV at the concentrations released from these ISMs against oral epithelial cell lines remains to be assessed, and the inherent acidity of the IBU and PA matrix system (pKa ∼4.4 and ∼4.8, respectively) may pose a risk of local tissue irritation or interference with wound healing in inflamed periodontal tissues. Furthermore, the in vitro release model does not replicate the dynamic hydrodynamic environment of a periodontal pocket, as salivary flow and GCF dynamics may accelerate matrix degradation and enhance drug diffusion under clinical conditions. These aspects are identified as priorities for future investigation in appropriate ex vivo or in vivo models before the clinical translation of these systems.

## Author Contributions

All authors contributed substantially to the study’s conception and design, data collection, analysis, and interpretation, as well as to drafting or critically revising the manuscript for important intellectual content. T.P. supervised the project and managed the research administration.

## Funding

This work was supported by Walailak University under the New Researcher Development scheme (Contract No. WU69221).

## Disclosure

All authors have reviewed and approved the final version for publication. The authors independently verified all information, data accuracy, and scientific interpretations and take full responsibility for the final content.

## Ethics Statement

The antimicrobial experiments described in this study were conducted in accordance with institutional biosafety regulations and approved by the Institutional Biosafety Committee (IBC) of Walailak University (Approval No. WU‐IBC‐68‐056).

## Conflicts of Interest

The authors declare no conflicts of interest.

## Data Availability

The data that support the findings of this study are available from the corresponding author upon reasonable request.
